# Clinical scientist led clinic in adult congenital heart disease – how to do it?

**DOI:** 10.1186/s44156-025-00097-w

**Published:** 2025-12-08

**Authors:** Dario Freitas, Mitch Fenn, Brian Campbell, Hannah Douglas, Stefano Svab, Harith Alam, Alessandra Frigiola, Yaso Emmanuel, Natali Chung

**Affiliations:** 1 Clinical Scientist in Cardiology, Adult Congenital Heart Disease Department, Guy’s and St Thomas’ Hospital, NHS Foundation Trust, London, UK; 2 Senior Principal Clinical Scientist in Cardiology, Cardiology Department, Imperial College Healthcare NHS Trust, London, UK; 3 Consultant in Cardiology, Adult Congenital Heart Disease Department, Guy’s and St Thomas’ Hospital, NHS Foundation Trust, London, UK

**Keywords:** Adult congenital heart disease (ACHD), Clinical Scientist-led clinic, Service innovation, Cost-effectiveness, Patient safety, Patient satisfaction

## Abstract

The increasing demand for adult congenital heart disease (ACHD) services requires innovative solutions to reduce waiting times and optimise patient care. This paper presents the implementation and development of an ACHD Scientist-led clinic at Guy’s and St Thomas’ NHS Foundation Trust, providing an alternative, cost-effective, and efficient service model. A comprehensive service audit identified challenges related to patient backlogs, clinic efficiency, and administrative coordination. Subsequent restructuring efforts, including an expanded appointment system, improved referral pathways, and enhanced clinical governance, led to a significant reduction in overdue appointments from 15% to 2%, despite an overall increase in patient numbers. Comparative cost analysis demonstrated that the Clinical Scientist-led model is more cost-effective than the traditional models. Additionally, patient satisfaction surveys and Consultant evaluations confirmed this model’s high quality and safety. These findings highlight the potential for broader adoption of Clinical Scientist-led clinics within the ACHD networks to improve accessibility, efficiency, and sustainability of care.

**Supplementary information:**

The online version contains supplementary material available at 10.1186/s44156-025-00097-w.

## Introduction

The primary goal of any healthcare service is to provide safe and effective patient care. Within the National Health Service (NHS) there has been an increasing emphasis on the provision of efficient and cost-effective services in diagnostic and patient management pathways that take advantage of a diverse and highly skilled workforce, without compromising patient safety. NHS England guidance on patient waiting times sets a maximum limit for non-urgent Consultant-led treatment pathways of no more than 18 weeks [1]. However, the COVID-19 pandemic had a negative impact on NHS waiting lists, with the number of people on general cardiology waiting lists reaching a record high of 408,061 in January 2024. This represented an overall increase in general cardiac waiting lists of 75% compared to February 2020, the month before the pandemic began, resulting in approximately 40% of patients breaching the 18-week target [2]. This led to the further development and expansion of existing non-Consultant led services in the NHS including Clinical Scientist-led services, particularly in valvular heart disease and stress echocardiography [3–5].

The current estimated prevalence of adult congenital heart disease (ACHD) patients is approximately 3000 per million population [6], and it is now well established that the care of congenital patients is a lifelong process that requires pathways and strategies planning for surveillance and interventions when needed, leading to an ever-expanding demand on adult congenital heart services [6]. The ACHD Physiologist-led clinic had been in place at Guy’s and St Thomas’ Hospital NHS Trust (GSTT) for several years before the COVID-19 pandemic. However, the ongoing increase in patient numbers, combined with the additional backlog caused by the pandemic, urged the need to enhance the existing service model. Building on the structure and governance of other existing Scientist-led services at GSTT [5], we adapted and expanded the clinic using a modernised Clinical Scientist-led model. A review of current literature regarding Clinical Scientist-led services did not reveal any other service of this type in relation to ACHD. In this paper, we describe how the Clinical Scientist-led model was developed to address the significant increase in waiting times in a safe and efficient structure that clearly demonstrated improved service delivery for ACHD patients.

## Background and rationale

In 2015, the British Cardiac Society (BCS) and the Society for Cardiac Science and Technology (SCST) published a joint strategic review of Cardiac Physiology services in England, which highlighted the need and value of non-Consultant led services [7]. The modernisation of scientific careers and access to professional registration as a Clinical Scientist with the Health and Care Professions Council (HCPC) through the Scientist Training Programme (STP) or the STP equivalence process has enabled the development of autonomous practice with appropriate accountability [7]. In 2017, the multidisciplinary framework for advanced clinical practice model in England emphasised the need for new ways of working in healthcare that could deliver solutions to meet the population’s changing needs [8].

The development of an ACHD non-Consultant led clinic at GSTT first started in 2014. Various service models were reviewed and explored. These included ACHD Nurse-led clinics with echocardiography support from Physiologist/Scientists and the Physiologist/Scientist-led clinics. The latter were initially limited by the availability of appropriately qualified and trained staff. The expansion of the ACHD Physiologist/Scientist team with full accreditation in congenital echocardiography created an opportunity to re-evaluate these service models with a focus on a more sustainable structure, hence a full review of the service was undertaken between September 2022 and March 2024. The primary aims of this review were to appraise the service requirements, determine the feasibility of an ACHD Scientist-led clinic service with appropriate quality assurance processes, and ensure the model was of sufficiently high-quality. Feedback from both patients and service users were obtained. Data collection was split into two stages. We first audited the service between September 2022 and March 2023, and then re-audited between March 2023 and December 2024 to evaluate the impact of changes implemented to our clinical practice.

## Clinical practice overview

### Initial service model

Patients within the ACHD service were first seen by a specialist Consultant Cardiologist, who then referred appropriate patients, based on the underlying pathology and the initial assessment, to the Clinical Scientist-led service for longer term surveillance (see appendix 1).

Initially, the Physiologist/Scientist-led clinic occurred every second week, with a total of four one-hour slots. Each patient would have a 12-lead electrocardiogram (ECG) and basic observations in the outpatient department on arrival, performed by a cardiographer/healthcare assistant. The Physiologist/Scientist would then take the clinical history based on a proforma (see appendix 2), perform a cardiovascular examination and a transthoracic echocardiogram. The patient would be informed by the Physiologist/Scientist about the results of the assessments. Appropriate advice on lifestyle, endocarditis prophylaxis, and family planning that had previously been given at the initial Consultant review would be reinforced, along with a follow-up surveillance plan. The outcome of the clinic would then be communicated to the general practitioner via a clinic letter and a copy sent to the patient and the referring ACHD Consultant. Each patient would be given contact details for the ACHD Clinical Nurse Specialist (CNS) team to discuss any further concerns. In the event of significant abnormal echocardiographic findings or clinical change, the escalation protocol was to contact the referring ACHD Consultant or the ACHD Consultant on-call, according to their availability and the clinical urgency.

## First audit

The existing clinic patient database of 345 patients was reviewed and validated between September 2022 and January 2023. The following issues were identified:

- 52 patients (15%) had unscheduled overdue appointments from the time of the last review;
- All available appointment slots until the end of 2023 were full, and it was not possible to schedule the overdue patients;
- The rate of patients who did not attend (DNA) the clinic was 20%.

Further evaluation revealed administrative issues at the very start of the clinic pathway with referrals from Consultants being sent to the administrative team on some occasions and on others occasions directly to the Physiologist/Clinical Scientist team, resulting in delays in action. Upon review, it became clear that the patient’s referral pathway and communication between the teams involved needed to be clarified and streamlined.

The composition of the patient cohort and the appropriate follow-up periods for each pathology were not clearly defined (generally 2 years), resulting in inconsistencies in patient follow-up plans. Discrepancies in follow-up intervals, particularly for mild lesions, have been discussed in the literature, highlighting differences between current clinical practice and published guidelines [9].

Until 2022, the clinic provision model was varied, alternating with support from the ACHD CNS team, partially due to there being only one appropriately trained Physiologist/Clinical Scientist who could undertake all aspects of the clinic independently.

While the new training plan for Clinical Scientists with respective competencies included full congenital echocardiography accreditation and a clinical assessment course, there was no fully documented internal signing-off process/assessment in place.

Whilst a copy of all clinic letters was sent to the original referring ACHD Consultant, there was no formal audit process seeking the Consultant views on the quality of the service. Moreover, no feedback was sought from the clinic patients themselves.

Finally, the “value for money” of the different service models was unclear. This was considered to be an essential component in the assessment of the efficiency of the different models: traditional ACHD Consultant-led, CNS-led or Clinical Scientist-led clinics.

## Clinical practice changes

Several revisions of the ACHD Scientist-led model were implemented to improve the clinic workflow.

## Clinic structure and administration

While the one-hour slots were kept to ensure adequate time for a thorough patient assessment, the booking process was amended by asking the patient to arrive 30 minutes earlier to facilitate the initial clinical observation and a 12-lead ECG in the outpatient department prior to the appointment.

The initial audit indicated a clear need to increase capacity. Therefore, we increased the number of slots from 4 to 7, still on a fortnightly basis, starting in March 2023.

To address potential gaps in the patient referral pathway and improve communication across the ACHD multi-disciplinary team, a weekly meeting was created and included Secretaries, the Assistant Service Manager, the lead Clinical Scientist, and the Lead Consultant when available. The meeting reviewed referrals and identified any booking problems with input from the wider team. The team reviewed the high DNA rate and initiated two specific strategies:

1. The secretaries would phone the booked patients up to a week before, to remind the patient about their appointment.
2. If the patient said they were not available, the secretary would proactively fill the slot with another patient to ensure the clinic capacity was maximised.

## Clinical governance and sustainability

**Fig. 1:**
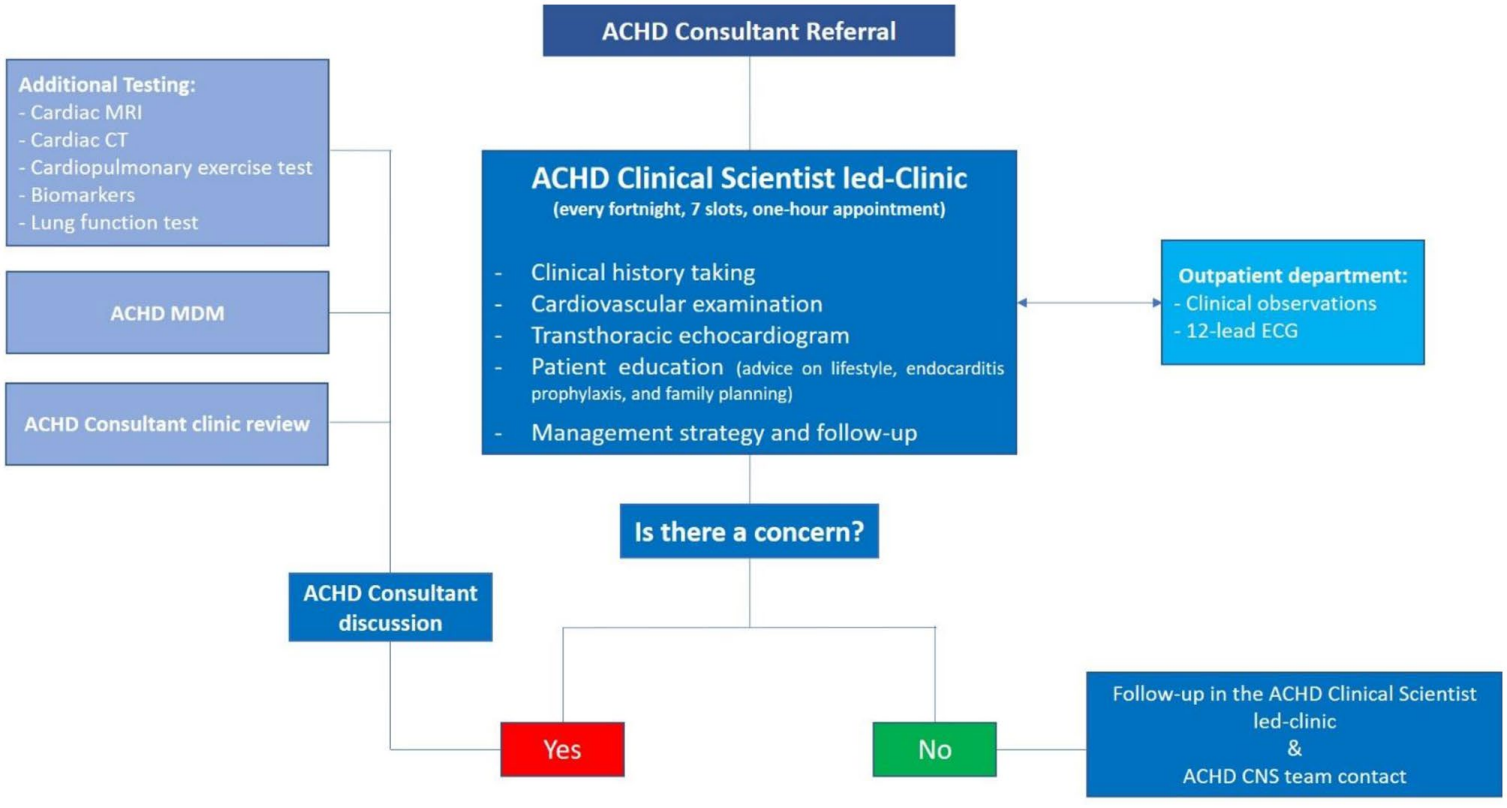
Organisation of the current ACHD clinical Scientist-led service (MRI-Magnetic resonance imaging, CT- Computed tomography, MDM- Multi-disciplinary medical decision meeting, ECG - Electrocardiogram, CNS - Clinical nurse specialist, ACHD - Adult congenital heart disease)

New documents were created to standardise the patient assessment. A clinical assessment proforma (see appendix 3) and outcome letter template (see appendix 4) were implemented with a focus on clinical history, symptoms, lifestyle, infective endocarditis prophylaxis, family history of congenital heart disease and screening advice, contraception and pregnancy recommendations relevant to the ACHD patient population. A clinic letter summarising relevant clinical examination findings, 12-lead ECG and echocardiography results, and clinical outcomes, including appropriate follow-up and contact information, was sent to the general practitioner and a copy to the patient. A copy of the clinical questionnaires was kept in the hospital system. For routine follow-up appointments, we deemed it no longer necessary to copy the letter to the referring ACHD Consultant. If there were any concerns or potentially significant new findings, these would be discussed with the referring ACHD Consultant, after which additional testing and/or review at the ACHD multi-disciplinary medical decision meeting or indeed transfer back to the Consultant-led clinic are possible outcomes (see Fig. 1). As before, each patient would be given contact details (utilising a QR code option) for the ACHD CNS team, to discuss any further queries or concerns in between each clinic appointment.

**Fig. 2:**
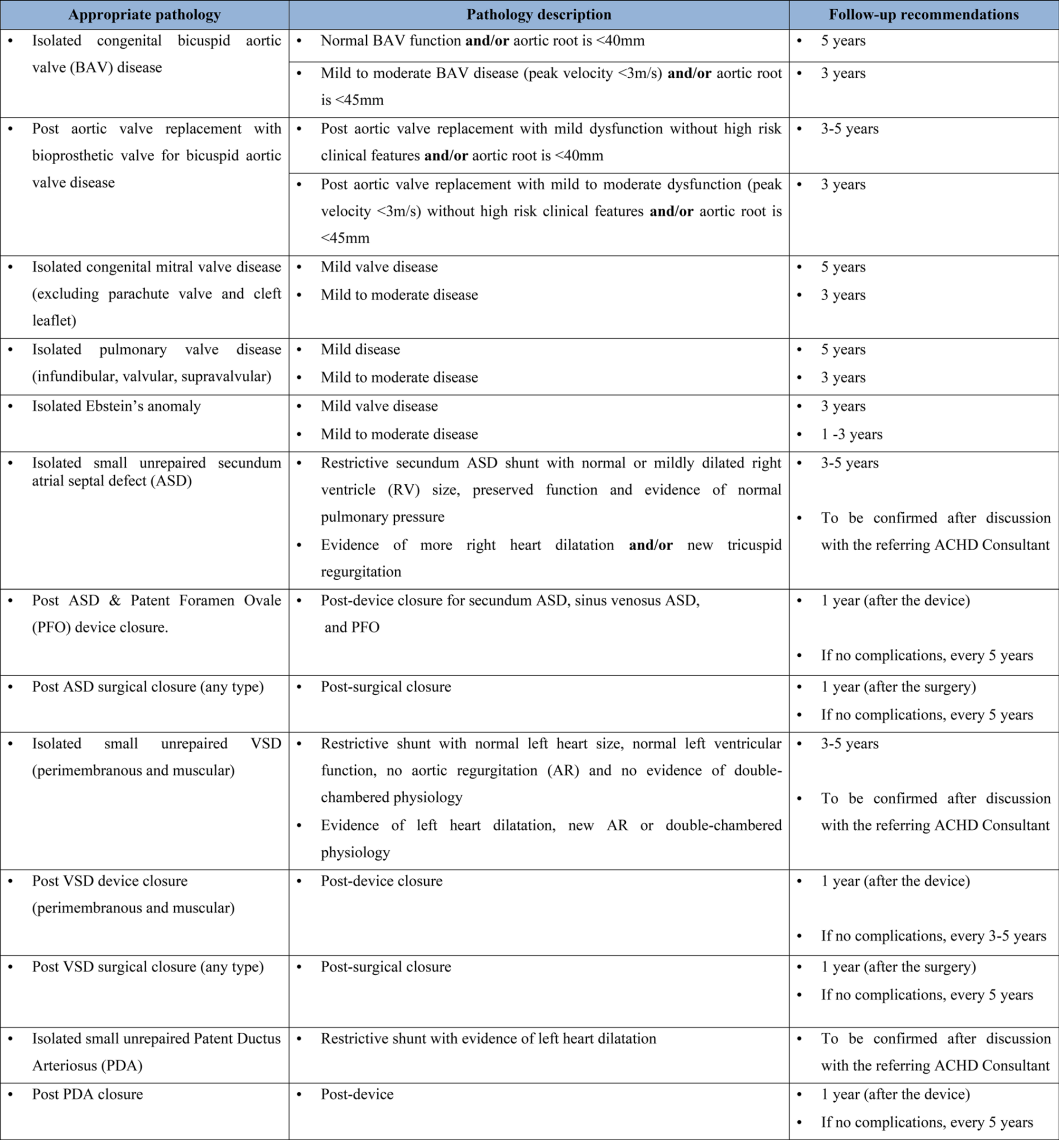
Current list of appropriate pathologies seen in the ACHD Scientist-Led Clinic. Abbreviation: BAV - Bicuspid aortic valve, ASD - Atrial septal defect, RV - Right ventricle, PFO - Patent foreman ovale, ACHD - Adult congenital heart disease, VSD - Ventricular septal defect, AR - Aortic regurgitation, PDA - Patent ductus arteriosus

The list of the appropriate pathologies seen in the clinic was further refined and a table developed to include mild structural and functional congenital cardiac conditions as described in the 2020 European Society Cardiology ACHD guidelines [10]. Additional pathologies outside of the 2020 ACHD guideline for clinically stable patients were also added to the list based on a consensus of the ACHD Consultant and Clinical Scientist groups. The pathologies and appropriate follow-up recommendations are described in Fig. 2.

Training to increase the number of Clinical Scientists was given priority to facilitate the increase in the number of clinic slots. The Clinical Scientist training requirements were defined as:

- Congenital heart disease echocardiography board accreditation (European Association of Cardiovascular Imaging or British Society of Echocardiography);
- Successful completion of an external clinical assessment course;
- An internal sign-off after completing a logbook of 25 patients’ assessment during clinics/ward rounds with appropriate clinically trained staff.

**Fig. 3:**
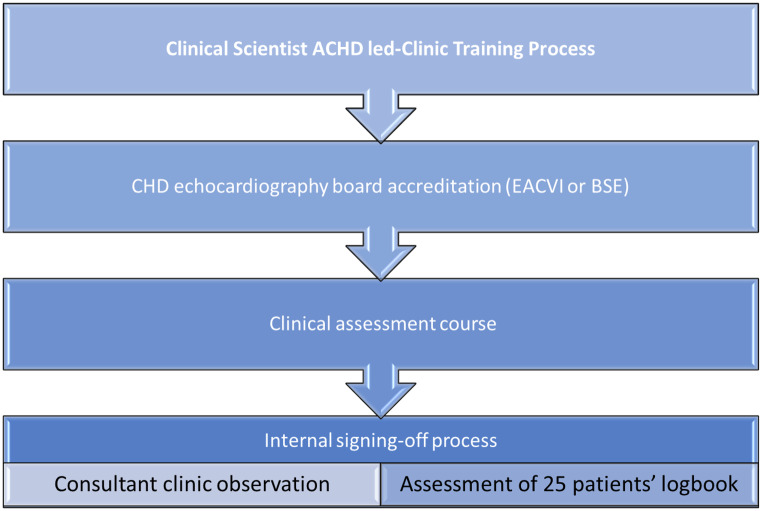
ACHD clinical scientist-led service training and sign-off process/assessment. Abbreviation: EACVI - European Association of Cardiovascular Imaging, BSE - British Society of Echocardiography

In addition to the Lead of the ACHD echocardiography service, another Clinical Scientist within the ACHD echocardiography team with experience in Valve clinics and also congenitally accredited joined the team, providing a second staff member to further improve the sustainability of the service. Finally, a third Clinical Scientist with expertise in ACHD echocardiography was trained by the team (see Fig. 3).

## Second audit

### Clinic structure and administration

**Fig. 4:**
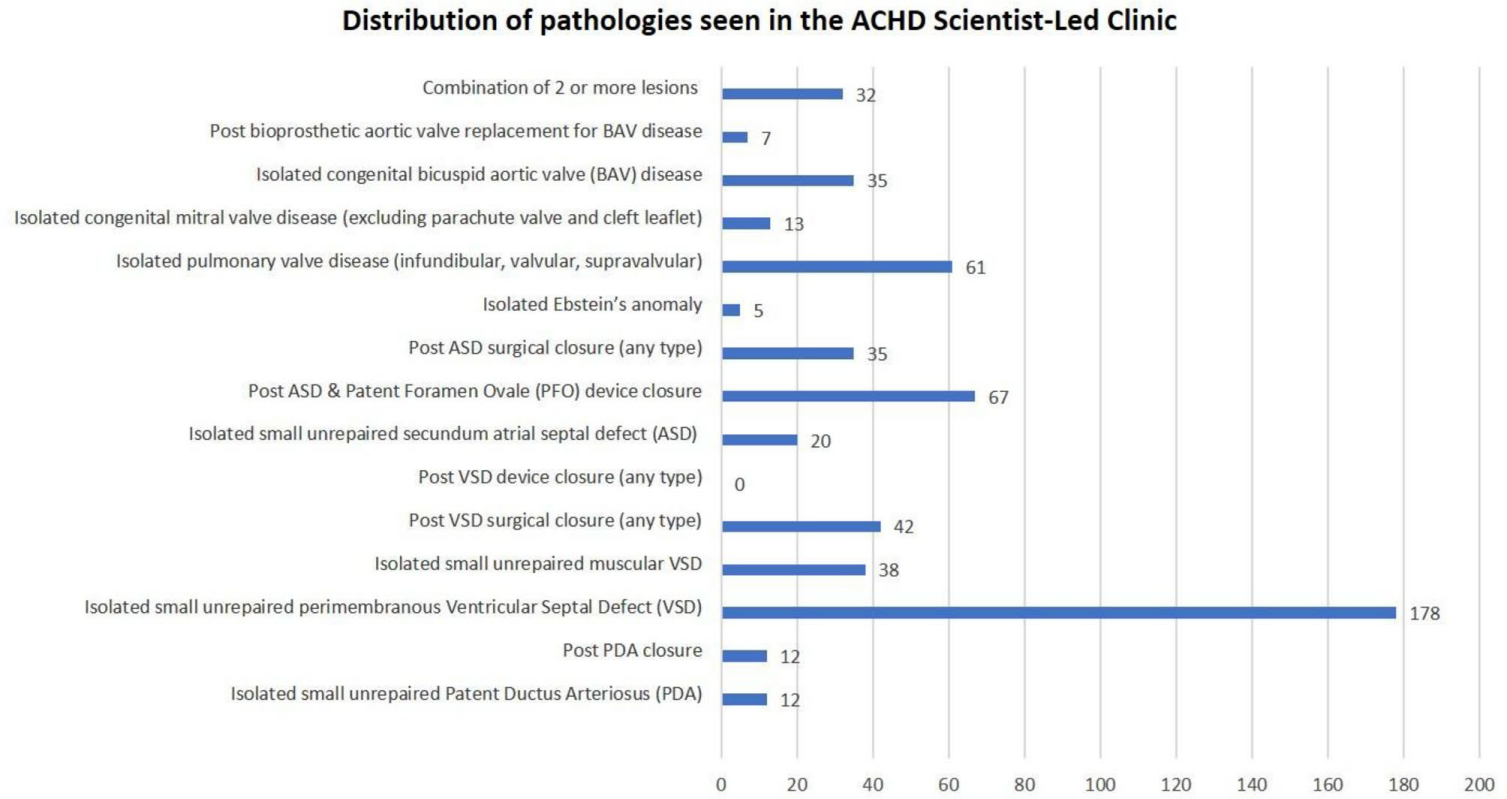
Distribution of pathologies seen in the ACHD Scientist-Led clinic. Abbreviation: ACHD - Adult congenital heart disease, BAV - Bicuspid aortic valve, ASD - Atrial septal defect, PFO - Patent foreman ovale, VSD - Ventricular septal defect, PDA - Patent ductus arteriosus

After implementing all the structural changes, the patient database was reviewed to understand the overall impact on the service between March 2023 and December 2024. This proved to be challenging as the Trust transitioned to a new patient electronic record in October 2023, resulting in a team effort to undertake manual data validation for each individual patient. Overall, there was an increase from 345 to 557 (38%) patients within the Clinical Scientist-led clinic. Out of a total population of 557 ACHD patients, 340 were females (61%), 217 were males (39%), with ages varying between 18 and 85 years old, with a mean age of 38 ± 6 years. There was a heterogeneous distribution of pathologies, with the greatest number of patients, *n* = 178 (32%) presenting with isolated unrepaired perimembranous ventricular septal defect (see Fig. 4).

With regard to waiting times changes, 58 patients (10%) were scheduled to be seen by the end of 2024, and 490 patients (88%) were on the waiting list due to be seen between 2025 and 2029. Only 9 patients (2%) breached their planned review date in the second audit period. Thus, despite a significant increase in the number of referrals made to the clinic, which reflects the continuous expansion of the ACHD population and the Consultant’s engagement with the service, the number of overdue patients fell from 15% to 2%, reflecting the excellent positive impact of the service expansion process and the changes that were implemented.

## Clinical governance and sustainability

**Fig. 5:**
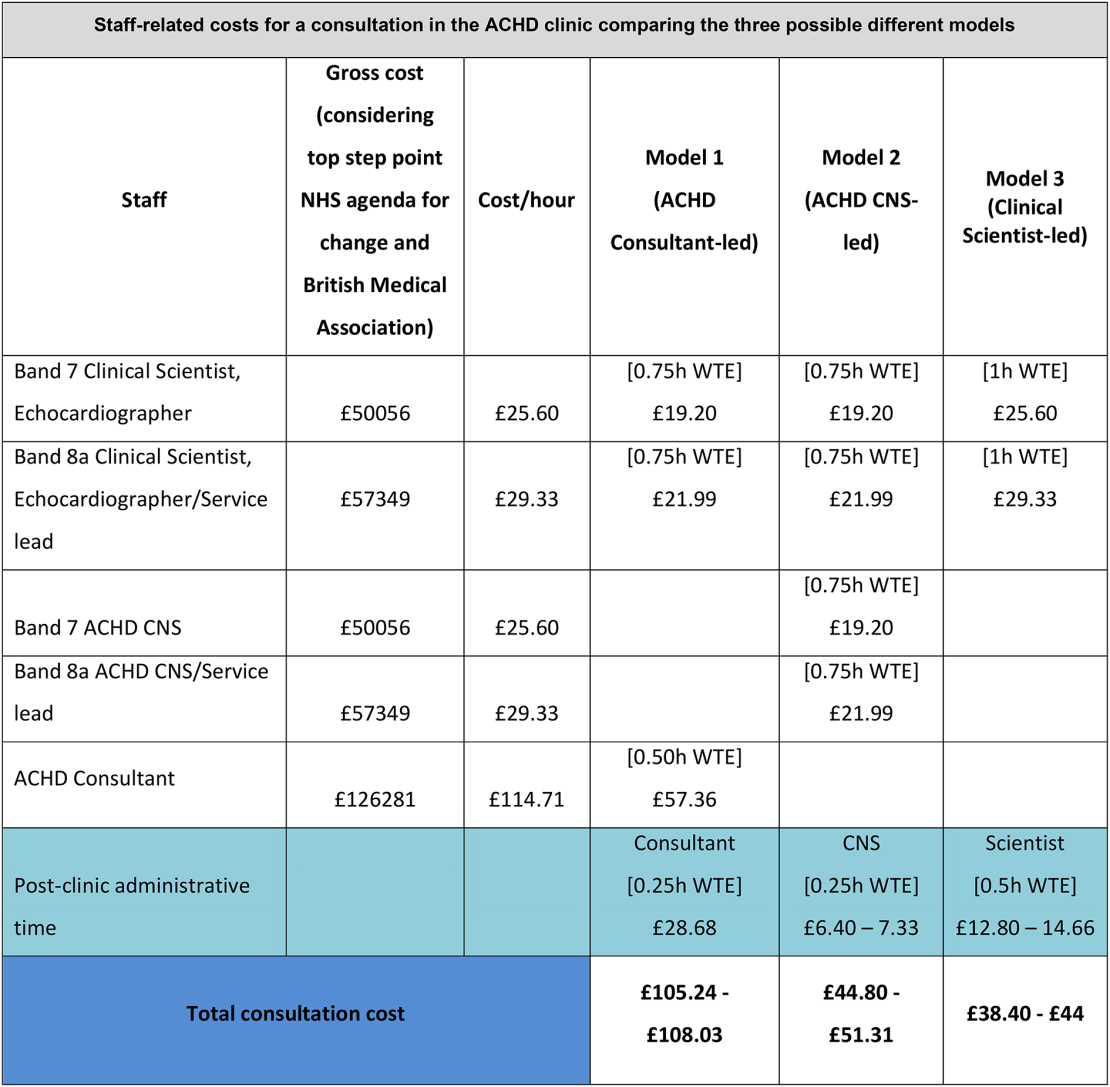
Value for money: staff-related costs for a consultation in the ACHD clinic

A review of costs of the three different ACHD service models was undertaken (see Fig. 5):

- Model 1- the ACHD Consultant seeing patients with echocardiography support from an ACHD Physiologist/Scientist;
- Model 2 - the ACHD CNS seeing patients with echocardiography support from an ACHD Physiologist/Scientist;
- Model 3 - the ACHD Clinical Scientist seeing patients and performing the echocardiography study.

The review took into consideration the time allocation according to the clinic practices at GSTT and the basic band scale according to the staff grade in the NHS in the United Kingdom in 2024. Consultant time allocation per patient was 30 minutes average in clinic and 15 minutes post-clinic administrative time plus 45 minutes Physiologist/Scientist scanning and reporting time. CNS time allocation was 45 minutes in clinic and 15 minutes post-clinic administrative time, plus 45 mins Physiologist/Scientist scanning and reporting time. Clinical Scientist time allocation was 60 minutes in clinic and 30 minutes post-clinic administrative. Expressing the staff requirements as working time equivalent (WTE), 15 minutes will correspond to 0.25 h WTE, 30 minutes will correspond to 0.50 h WTE, 45 minutes will correspond to 0.75 h WTE and 60 minutes to 1 h WTE. The analysis indicated that the ACHD Clinical Scientist-led service is 59% cheaper than a Consultant-led model and 15% cheaper than a Nurse-led model (see Fig. 5).

A patient survey was carried out between January and March 2024 to find out the patient’s experience with regard to their treatment and care during their clinic visit. The survey consisted of eight statements with six possible answers and additional space for comments (see appendix 5). Of the 20 participants (representing approximately 5% of the clinic population by then), 12 were women, and 8 were men. Most patient feedback was positive with 95% satisfaction, as shown in appendix 5. The survey results were helpful in understanding that patients were satisfied with the level of care provided and, above all, engaged with the service by suggesting what could be improved. That set the stage for creating a future patient group representative to promote the service’s development in the near future.

Finally, regarding clinical efficacy, an audit of the clinical assessments performed by the Scientist team was carried out on 20 patients between March 2023 and March 2024 (representing approximately 5% of the total clinical population) through evaluation by an ACHD Consultant. A clinician-reported outcome measures form was created among the service stakeholders, with two main questions, yes or no answers, and space for comments. The questions were as follows:

- Is all the relevant patient data included and well described to allow a full assessment of the congenital heart disease condition?
- Is the patient’s current clinical management and follow-up plan consistent with what should be expected for the congenital heart disease condition?

After analysing the 20 cases identified for pilot data, the ACHD Consultant’s conclusion mentioned that the criteria identified in the agreed standard operating procedure were achieved. The letters were high quality and included relevant clinical information while remaining succinct and easy to read. Two specific areas were identified for further improvement: more detail on awareness of endocarditis symptoms, and expansion on advice regarding family planning and management during pregnancy. These suggestions were subsequently incorporated into the clinical assessment documentation.

## Conclusion

The waiting times for the ACHD patient cohort increased significantly during the COVID-19 pandemic. The results of our comprehensive audit and re-design of the ACHD Clinical Scientist-led model showed that it is possible to achieve a high-quality service that is both efficient and cost-effective.

The clinical skills and autonomy to undertake and interpret cardiac examinations safely in an ACHD Clinical Scientist-led service was demonstrated with clear practice standards improved by audit and appropriate clinical governance. Furthermore, investing in the Healthcare Science workforce has anecdotally improved job satisfaction and may help with the retention of skilled staff in a profession that currently has a national shortage. While BSE reports have alluded to further training being viewed positively by staff in terms of retention, we propose that a specific accreditation pathway in advanced clinical practice could be developed to achieve this goal.

Service evaluation and development of new pathways supported by audit principles and quality of care measurements allow a complete view of clinical services in the NHS and are crucial to promoting appropriate governance and high-quality service development. With the appropriate support from all the different service stakeholders in the ACHD team, Clinical Scientists can pursue advanced clinical practice with a high degree of autonomy within a specific range of ACHD pathologies.

These results were limited to a level 1 centre within an ACHD Network, and it is unclear how this service pathway would work in level 2 and level 3 centres where the role of the Clinical Scientist may not be as well recognised or structured. A potential option resulting from this service evaluation is that wider ACHD network centres could benefit from expansion of Clinical Scientist-led ACHD level 1 services to facilitate a bespoke model of training and support for the local teams. Despite the possible, such efforts will enable better access to standardised healthcare across the network and advance the horizontal integration of services needed to manage ACHD patients.

Finally, the benefit of audit was clearly demonstrated and so it should be regularly undertaken to ensure continuous improvement and service sustainability.

## Electronic supplementary material

Below is the link to the electronic supplementary material.

Supplementary material 1

## Data Availability

No datasets were generated or analysed during the current study.
