# Supplementary material for: Clinical scientist led clinic in adult congenital heart disease – how to do it?
Source: Echo Res Pract. 2025 Dec 8;12:32. doi: 10.1186/s44156-025-00097-w (PMC12683812; doi:10.1186/s44156-025-00097-w)
Supplement: Supplementary file 1 — Supplementary material 1 [file 44156_2025_97_MOESM1_ESM.docx]

**Appendix 1 – Initial list of appropriate pathologies seen in the ACHD Scientist-Led Clinic**

**
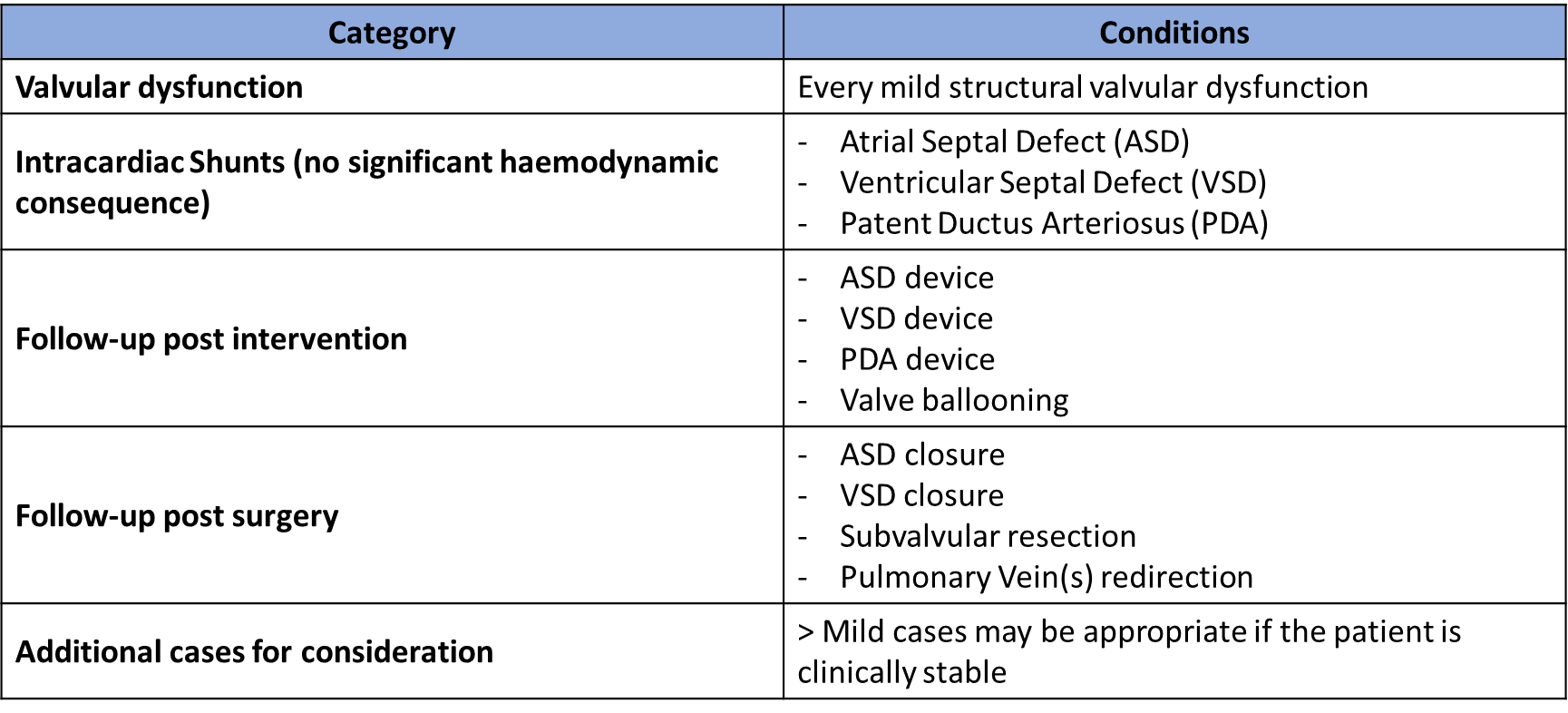
**

**Appendix 2 – Initial clinical history taking questionnaire template / letter**


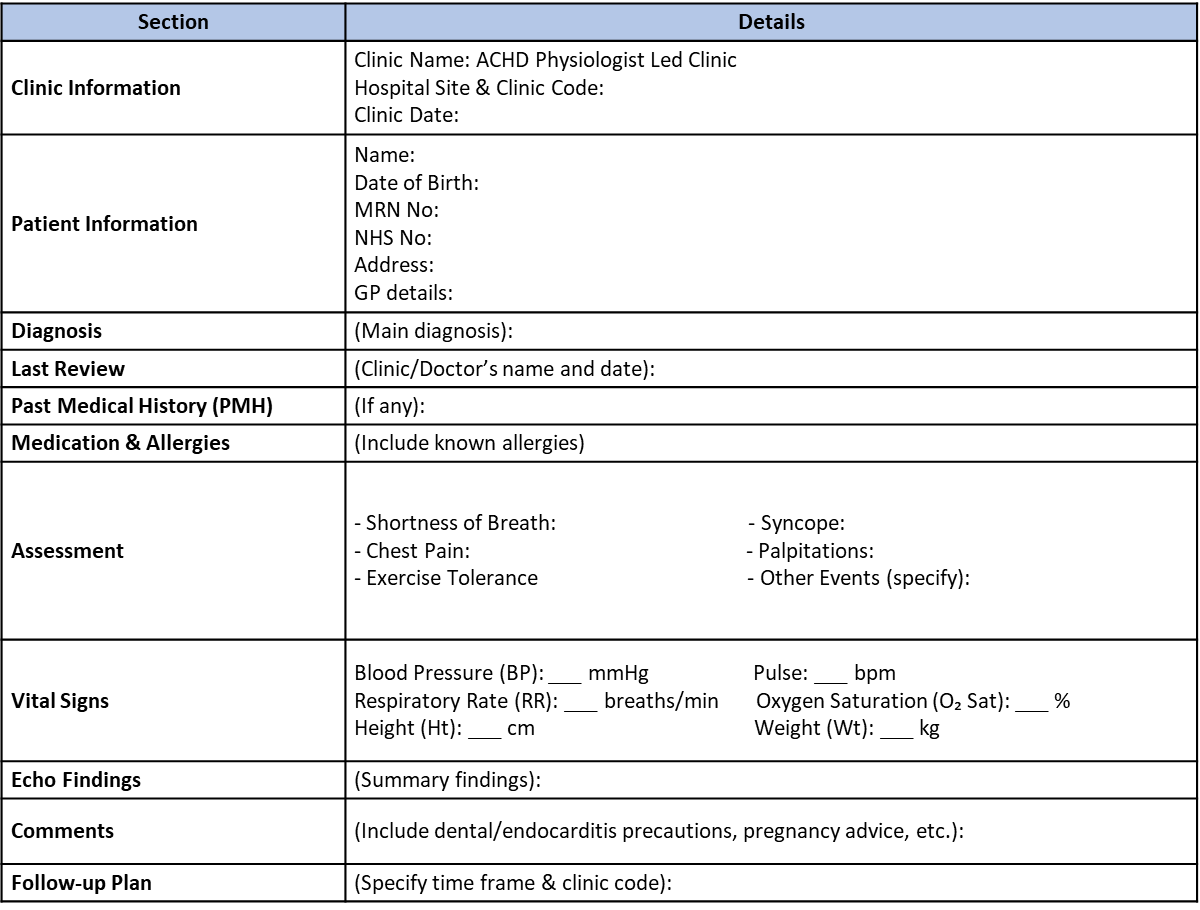


**Appendix 3 – Current clinical history taking questionnaire template**

**Clinic date and hospital site:**

**Patient details:**

**Diagnose(s):**

**Previous Procedure(s):**

**Medication (s):**

**Allergy (s):**

**Occupation/Part-time:**

**Exercise (activity, distance) and tolerance:**

**Chest pain/ Tightness** (**S**ite, **O**nset, **C**haracteristic, **R**adiation, **A**ssociated symptoms, **T**iming, **E**xacerbation, **S**everity):

**Syncope or Pre-syncope**:

**Dizziness or Lightheadedness:**

**SOB or Dyspnoea / Exertional SOB / Orthopnoea / Paroxysmal Nocturnal Dyspnoea:**

**Palpitations / Arrhythmia:**

**Oedema, location(s) and extension(s):**

**Other admission to Hospital:**

**Other on-going treatments:**

**NYHA class (I – IV):**

**Smoker (If quit ask when and how):**

**Drink habits (units of alcohol a week):**

**Recreational drugs:**

**Infective Endocarditis awareness (dentist visit, importance of a good dental hygiene at home, as well as the avoidance of tattoos and body piercing):**

**Contraception and advice:**

**Family history of CHD, screening and pregnancy plan (increased chance to transmit congenital heart disease to a future offspring; pregnancy counselling service is available if needed; pre-natal foetal should be performed on any future children):**

**Examination findings:**

**12 lead ECG findings:**

**Echocardiogram findings:**

**Other exams findings to check:**

**Follow-up plan and contact advice:**

**Appendix 4 – Current clinical letter template**

**Clinic date and hospital site:**

**Patient details:**

**Diagnose(s):**

**Previous Procedure(s):**

**Medication (s):**

**Allergy(s):**

It has been a pleasure to review Mr/Miss/Mrs in the Adult Congenital Heart Disease (ACHD) Scientist Led Clinic today. He/She is on this clinic under watchful surveillance for known *xx*.

Mr/Miss/Mrs is now *xx* years old and working as *xx*. He/She remains active doing *xxx* with a good exercise tolerance and no cardiac symptoms. There are no other medical problems that need to be reported and overall, he/she feels there has been no significant change since the last visit in *xx*.

He/She is a non-smoker who drinks *xx* units of alcohol a week. He/She keeps a good hygiene at home and attends the dentist regularly. He/She is aware of our advice about the importance of keeping yearly visits to the dentist, as well the importance to avoid any tattoos or piercings, body piercings, in way to prevent bacterial infection in the heart (endocarditis). We also gave advice about the signs and symptoms of a potential endocarditis infection.

*(Given the previous episode of infected endocarditis in xx, we would still recommend that he/she has antibiotic prophylaxis prior to any significant dental work)*

Miss/Mrs is taking/not taking contraception and is aware of our advice that she may have any type of contraception that she feels comfortable with, in any form / is recommended to have Progestin-only contraception.

*Miss/Mrs does not have family history of congenital heart disease and I explained that if she becomes pregnant there is an increased chance to transmit congenital heart disease to a future offspring. Therefore, a pregnancy counselling service is available and that a 20 weeks foetal scan is recommended on any future children. She understands and agrees with the plan.*

*Mr does not have family history of congenital heart disease. He also does not have family plans at the moment, however, I also explained that should this change, the future female partner should inform the antenatal team of his cardiac history and a 20 weeks foetal scan is recommended. He understands and agrees with the plan.*

**On examination** *(Example of a VSD patient)*

he/she looked well. Weight was *xx*kg and height *xx*cm. Pulse *xx*bpm, regular and strong. Blood pressure *xxx*/*xx* mmHg. Saturation *xxx*% on air. JVP not raised. No right ventricular heave or thrill. There 2 heart sounds and no murmur. There is a *xx* murmur, loudest at the *xx* left sternal edge. No evidence of oedema or hepatomegaly. Chest clear.

**12 lead ECG:** Sinus rhythm at *xx*bpm, normal axis. Normal intervals and QRS progression. No repolarisation abnormalities.

**Echocardiogram:** *(Example of a VSD patient)*

LV is normal size with *xx*mm. Normal volumes and normal function. Perimembranous VSD, peak gradient left to right above xxmmHg. *Xx* aortic regurgitation. RV is normal size and systolic function without evidence of RV outflow tract obstruction.

Overall, Mr/Mrs is clinically stable from cardiovascular perspective. We will arrange follow up with repeat echo in the ACHD Scientist Led Clinic in *x* years. In the meantime, he/she knows that our team can be contacted via the telephone numbers and email address above if there is any queries or concerns. In addition to that, I gave her/him the clinical review flyer with extra information about how to contact us in the meantime.

Yours sincerely,

**Xxx , Clinical Scientist in Cardiology, ACHD Department**

**Letter to GP. Copy to the Patient**

**Cc’ Dr Xxx, ACHD Consultant Cardiologist, ACHD Department (when required)**

**Appendix 5 – Patient survey results**

|  | **Strongly Disagree** | **Disagree** | **Neither agree nor disagree** | **Agree** | **Strongly agree** |
| --- | --- | --- | --- | --- | --- |
| I was treated in a friendly courteous manner |  |  |  |  | **20** (100%) |
| The Clinical Scientist was knowledgeable about my condition |  |  |  |  | **20** (100%) |
| My test results were clearly explained to me |  |  | **1** (5%) | **1** (5%) | **18** (90%) |
| I have confidence in the information and advice I have been given |  |  |  | **1** (5%) | **19** (95%) |
| The Clinical Scientist listened to my concerns |  |  |  |  | **20** (100%) |
| The follow up plan for my care has been clearly explained |  |  |  | **1** (5%) | **19** (95%) |
| I was happy with the amount of time I received for my review today |  |  |  | **1** (5%) | **19** (95%) |
| I am satisfied with the care I have received today |  |  |  | **1** (5%) | **19** (95%) |
| **COMMENTS:**  “Very friendly staff, make everything extremely comfortable”  “Very good service, happy with results”  “Friendly and professional. Very clear communicator”  “Excellent care”  “Thank you very much for a great service experience”  “I was really happy with everything today. Both Clinical Scientists were lovely”  “They knew about my condition and adjust their care to my knowledge”  “Superb service”  “My only comment would be for the clinical staff doing the initial observations to explain what was happening next. Also, this could be included on the appointment information to make my journey clearer” | | | | | |
